# Observation of Metacognitive Skills in Natural Environments: A Longitudinal Study With Mixed Methods

**DOI:** 10.3389/fpsyg.2019.02398

**Published:** 2019-11-01

**Authors:** María Consuelo Sáiz Manzanares, Miguel Ángel Queiruga Dios, César Ignacio García-Osorio, Eduardo Montero García, Jairo Rodríguez-Medina

**Affiliations:** ^1^Research Group DATAHES, Faculty of Health Sciences, University of Burgos, Burgos, Spain; ^2^Faculty of Education, University of Burgos, Burgos, Spain; ^3^Research Group ADMIRABLE, Department of Civil Engineering, Higher Polytechnic School, University of Burgos, Burgos, Spain; ^4^Research Group iENERGÍA, Department of Electromechanical Engineering, Higher Polytechnic School, University of Burgos, Burgos, Spain; ^5^Research Group DATAHES, Department of Education, Faculty of Education and Social Work, University of Valladolid, Valladolid, Spain

**Keywords:** metacognitive skills, quality, on-line evaluation methods, mixed methods, indirect observation, systematic observation, natural environments, personalized learning

## Abstract

Recent studies pointing to evaluation methods in natural environments suggest that their use in the analysis of metacognitive skills provides more precise information than the use of off-line evaluation methods. In this research, mixed methods are used over one academic year for the evaluation of the metacognitive skills that students of Secondary Education apply to solve physics problems. The objectives of this study are to analyze the use of metacognitive skills in natural environments and to study behavioral patterns of student learning through a longitudinal study. A total of 509 recordings of think-aloud protocols are analyzed through the categorization of the responses (liquefying) and the protocol of Van der Stel and Veenman for the analysis of the quality of metacognitive skills. Fewer conceptual errors and less uncertainty over vocabulary were noted during the academic year. Nevertheless, a degree of ambiguity persisted in the understanding of physics concepts. The metacognitive skills of Orientation and Planning were used more than any others. The technique of graph analysis is also applied, to establish the patterns of behavior of each student throughout the academic year. Different patterns were found, the analysis of which helped to identify academically challenged and at-risk students. The use of mixed observation techniques and graph analysis facilitated information on the pace of learning of each student. Future studies will be directed at proposals for the automation of these evaluation techniques in natural learning environments.

## Introduction

There is a need to investigate new forms of accessing knowledge in 21st century society. “How” to strengthen the use of metacognitive skills has been studied over three decades, because the use of those skills predicts 40% of student learning outcomes (Veenman, 2011). The studies of Veenman et al. (2014) provided guidelines on the use of an on-line evaluation method for the evaluation of metacognitive skills. Evaluation in natural environments is an implicit part of their method. Evaluation likewise permits the study of the quality use of those skills, rather than only their frequency of use. Hence, observational investigation is an important methodological tool that together with the latest technological advances in software engineering provides the researcher with data registers and data processing records.

### Quality Use of Metacognitive Skills

Flavell (1979), cited by Schellings et al. (2013, p. 964), defined metacognition as personal knowledge and regulation of cognitive activities during learning processes. These skills are related with the capability of learners to reflect on their own mental processes, permitting conscious and deliberate control of their cognitive processes. Veenman et al. (2006) understood metacognition as an agent of a higher order that supervises and directs the cognitive system whilst at the same time forming part of it. Those authors accepted the distinction between metacognitive knowledge and metacognitive skills. Following Schraw (1998, p. 114), the majority of investigators have distinguished between two components of metacognition: knowledge of cognition and self-regulated cognition. Knowledge of cognition refers to what individuals know, either of their own cognition or of cognition in general. It includes at least three classes of metacognitive awareness: declarative knowledge, procedural knowledge, and conditional knowledge. The first refers to knowledge of things (“What”), the second refers to problem-solving processes (“How”), and the third indicates why and when actions are taken. Likewise, on many occasions, learners are unaware that they are making use of metacognitive skills. That perception of their real use can be distorted by self-perceptive conditioners that limit the ecological validity that occurs when off-line methods are applied to the evaluation of metacognitive skills (Veenman, 2011). Hence, recent investigations have claimed that the most reliable form of analysis of metacognitive skills is during task completion in natural environments (Schellings et al., 2013). These methods have been referred to as on-line evaluation methods (Van der Stel and Veenman, 2014), because they are done during the implementation of a task and they do not imply introspective thought processes on task implementation, unlike off-line evaluation methods, e.g., questionnaires (Van der Stel and Veenman, 2014). The evaluation of metacognitive skills in natural (classroom) environments for those authors begins with the collection of information on the resolution of common learning tasks. Subsequently, that information, in the form of either audio or video recordings, is categorized by different criteria on task completion (correct, incorrect, etc.). Various studies (Schellings et al., 2013; Van der Stel and Veenman, 2014; Veenman and Van Cleef, 2019) proposed that it was clearly important to perform two types of analysis of the information that was recorded through on-line methods. On the one hand, they proposed the analysis of the frequency of use of metacognitive skills and, on the other, the study of the quality of those metacognitive skills. To do so, those authors proposed the use of evaluation protocols that cover a scale of 0–4, where 0 implies that the strategy is not employed and 4 implies a highly acceptable use of the strategy.

Likewise, Schellings et al. (2013, p. 965) according to the studies of Veenman and Beishuizen (2004) distinguished four types of metacognitive skills. The first type would be the skills of Orientation, which refer to activities that the learner undertakes to specify the demands of the tasks in cognitive terms, which precede the skills of Planning. Planning skills, in turn, refer to the establishment of the plan and its primary and secondary objectives. Subsequently the skills of Evaluation are developed, which refer to evaluation monitored throughout the problem-solving process: in other words, the supervisory strategies that the learner completes during implementation of the plan that is envisaged and the modifications, if necessary, that are introduced throughout the development of problem-solving phase. Finally, the fourth category consists of Information Elaboration skills, which imply mechanisms for reflection on the implementation of the task in relation to its objectives (Van der Stel and Veenman, 2014).

In this framework, the analysis of observational processes of the use of metacognitive skills is important, because it is directly related with the achievement of effective learning and with the acquisition and the use of procedural knowledge (Reoyo et al., 2017). That knowledge is related with the use of self-regulation strategies and with the use of the Planning metacognitive skills (Sáiz and Montero, 2015). The measurement of metacognitive skills must, for that reason, be as systematic and precise as possible and on-line methods of evaluation must be applied, because they facilitate the teacher with information on the problem-solving process in the “here and now”. The teacher will therefore be able to adjust curricular practice to the learning characteristics of the students. This adjustment will foreseeably increase effective student learning. The methods of on-line evaluation consist of recording student actions and verbalizations for their analysis using protocols for the evaluation of quality in the use of metacognitive skills (Veenman et al., 2014; Sáiz and Queiruga, 2018; Veenman and Van Cleef, 2019).

Another important variable in the study of learning processes is to consider the characteristics of the subject matter to be learnt. In particular, the results of investigations on the learning of physics suggest that the use of metacognitive skills (Orientation, Planning, Evaluation, and Elaboration) appears to be subordinate to the perceived difficulty that the student has of grasping the concept in the mind. Likewise, when beginning to learn scientific knowledge, it has been noted that the comprehension of many concepts is ambiguous (Pozo, 1994). The resulting hypothesis is that perhaps the acquisition of the different metacognitive skills (Orientation, Planning, Evaluation, and Elaboration) is not homogeneous (Sáiz and Queiruga, 2018). In fact, when the teacher initiates instruction, doing so will guide student learning toward the use of the Orientation and Planning metacognitive skills. These are initially necessary and refer to declarative knowledge (“What”) that is related to the previous knowledge that the learner has when facing a new task. In fact, the studies of Taub and Azevedo (2019) have detected that learners with better levels of previous knowledge activate more complex metacognitive skills; in so far as the use of the Evaluation and Elaboration metacognitive skills occur at superior stages of the problem-solving processes (Azevedo et al., 2011; Sáiz and Montero, 2015). These metacognitive skills are related with procedural knowledge (“How”) and conditional knowledge (“Why” and “When”). It is related to the proposal by Veenman et al. (2006) directed at training for increased use of metacognitive skills through the use of the questions What to do, When, Why, and How to do it (WWWH). Recent studies (Sáiz and Marticorena, 2016) have indicated that these sorts of skills are where the greatest differences between students are detected, in both secondary education and at university. It is likewise where most difficulties have been found in the implementation of Self-Regulated Learning (SRL) programs (Núñez et al., 2011).

### Analysis in Natural Environments of the Use of Metacognitive Skills

The use of observational methodology in natural environments will facilitate the reconstruction of situations and the microanalytical analysis of WWWH the events occur. In addition, observational methods will facilitate the study of the order in which the events occur in different situations, which assigns an ecological niche to an individual. However, both quantitative and qualitative techniques of analysis have to be applied, in order to study these patterns in a rigorous manner, through the use of mixed investigation methods (Johnson et al., 2007; Bakeman and Quera, 2011; Anguera et al., 2018a). That methodology can be applied by using direct observation (direct analysis in natural environments) and indirect observation (interviews, narratives recorded in natural scenarios) or both. Indirect observation requires the transcription of verbal material (audio and video recordings) from which the sequence of events and their duration are evident. Subsequently, this information must be “liquefied” (Anguera et al., 2018a). To do so, the information recorded in natural environments must be systematically transformed into coded matrices that are suitable for quantitative methods of analysis. This technique permits the systematic analysis of many details that occur in the events (Anguera et al., 2017b). The information that is obtained can be used in a qualitative form, for example, by using ethnographic methods (narrative studies), and, in a quantitative form, after coding the data. On this latter point, there are four steps to systematic observation: (a) formulation of a research question; (b) data collection in natural situations (audio or video recordings of verbal behavior in multiple dialogs can be used); (c) processing these data to study them applying both qualitative and quantitative methods; and (d) communication of the results.

The use of mixed investigation methods is therefore full of challenges, especially in indirect observation. As previously indicated, that observation implies the use of resources that follow a rigorous transformation of the information into coded matrices. From that paradigm, indirect systematic observation is in itself converted into a mixed method (Anguera and Hernández-Mendo, 2016; Anguera et al., 2017a). Following Anguera et al. (2018b), the mixed methods of indirect observation have to follow the steps outlined below to complete the categorization and to liquefy the data:

1. Specification of the dimensions of the study. These dimensions are taken from the theoretical foundation of the framework of the study.
2. Specification of the segmentation criteria to create the textual units, a process called “unitizing,” in which information that is not relevant to the study is omitted. Krippendorff (2013) recommended various segmentation criteria, among which the most widely employed has been the interlocutory criterion that takes each sentence uttered by each participant as a unit of analysis.
3. Construction of an indirect instrument of observation, for which a referent is needed to conduct the analysis that includes the codes of observation. This type of instrument can include rating scales. In addition, the units of observation have to be very precisely defined, to establish with great precision where to include or not to include a particular conduct that is observed, establishing categorical clusters, and eliminating categorical haziness. The categorical systems therefore have to comply with the principles of exhaustiveness and mutual exclusivity.
4. Codification of the information: the investigators have previously to decide how to register the information, for which purpose they have to use carefully selected sources, and how to organize it properly, for which purpose they have to code it using a formal system. On this point, the use of software packages such ATLAS.ti (2018), MAXQDA (2018), and NVivo (2018) can be helpful, among others.
5. Quantitative processing of coded matrices: implies rigorous control over data quality. To do so, the categorization has to be done using more than one observer, and an indicator in agreement with inter-evaluators must subsequently be found (Casarrubea et al., 2018). Different indicators can be applied, for example, Pearson’s contingency coefficient *C* (López-Roldán and Fachelli, 2015, pp. 28–29) (expressing the intensity of the relation between two or more qualitative variables, which is based on the comparison of the sequences of two characteristics with the expected frequencies). This coefficient is computed by calculating χ^2^, adding the categorizations of the two judges in the analysis of the responses of the subjects in all the units of analysis and then eliminating any empty categories (see Eq. 1).
  (1) C=√(χ2/(N+χ2)
  where *N* is equal to the number of judges.

Pearson’s coefficient of contingency establishes the association between two nominal variables, if the number of rows and columns is very high. Pearson considered the coefficient as a nominal approximation of the product-moment correlation for the interval variables (cf. Meijer et al., 2012 cited by Schellings et al., 2013, p. 974).

It is also common to employ the Alpha (α) coefficient of Krippendorff (2013) that analyses the agreement that the evaluators reach on the categorization of different units of analysis (see Eq. 2).

(2) α=1-D⁢o/D⁢e

Do is equal to the observed disagreement and De is equal to the expected disagreement.

Likewise, indirect observational methodology is currently used in natural teaching and learning environments, and in health to follow different therapies, among others (Curry and Nunez-Smith, 2015; Winter, 2018).

In this study, indirect observational methods were applied to natural environments directed toward the study of quality use of metacognitive skills in the learning of physics concepts. The classification of Veenman (2011) (Orientation, Planning, Evaluation, and Elaboration) and the protocol of Van der Stel and Veenman (2014) were used: (a) to analyze the types of responses from students during the resolution of physics problems over one academic year; (b) to analyze the quality of the metacognitive skills that students use over one academic year; (c) to study whether a relation exists between the use of metacognitive skills during the resolution of physics problems over one academic year; (d) to study the patterns of use of the metacognitive skills of each student over one academic year.

## Materials and Methods

### Design

A nomothetic multidimensional and prospective longitudinal design was applied (Anguera et al., 2001).

### Participants

The criteria for inclusion in this study were as follows: the participants had to be students from Secondary Education studying physics among other subjects. In contrast, the exclusion criterion was that the students had not been diagnosed with intellectual disability according to the criteria of DSM V. Convenience sampling was used for the choice of center and the sample. A total of 10 students participated, six men (*M*_age_ = 17.17 and *SD*_age_ = 0.41) and four women (*M*_age_ = 17 and *SD*_age_ = 1.16), a teacher specializing in physics teaching, and an external expert evaluator in techniques of qualitative analysis. A longitudinal study was performed over on academic year. 19 audio sessions were recorded (one per thematic sub-unit) that were divided into 509 textual units, one for each sentence uttered by each student, following the recommendations of Krippendorff (2013) (see section “Procedure”).

### Instruments

1. An SRL program of physics concepts: the program had 10 thematic units that were in turn sub-divided into 19 thematic sub-units that covered physics units from the final years of the Secondary Education curriculum. The contents covered in each thematic unit and sub-unit are described in Table 1. The following aspects were considered in each sub-unit: analysis of previous concepts (before the start of each thematic unit, a scale of evaluation was applied on the previous knowledge of the unit), unit objectives (indicate the learning objectives of the unit, in other words, what the student is expected to have acquired by the end of the thematic unit); evaluation indicators (that refer to the acquisition of the objectives for each unit); tasks (tasks to support the acquisition of the concepts of the unit); materials (referring to the materials that are necessary to work through the activities proposed in each unit); and generalization activities [referring to activities similar to those worked in the unit, but with a different presentation structure (Queiruga et al., 2016, pp. 309–455)].
2. Audio recordings of protocols (one for each sub-unit, 19 in total): the average duration of each recording session was 38 and 760 min were recorded. The average number of minutes per register by thematic unit was 33.42 and the standard deviation was 4.80.
3. “Protocol for the analysis of the quality of metacognitive skills” of Van der Stel and Veenman (2014) and Queiruga et al. (2016, pp. 456–538) applied to the learning of physics: this instrument includes the guide for observing the quality use of the four metacognitive skills (Orientation, Planning, Evaluation, and Elaboration), which measures quality use on a scale of 0–4, from “No use of the strategy” to “Use of the strategy in the most acceptable way.”

**TABLE 1:** List of contents by thematic unit and Sub-unit of the SRL program of physics concepts.

**Thematic unit**		**Thematic sub-unit**
Unit 1	Scientific methods. Physical magnitudes	(1.1) The scientific method
		(1.2) Preliminary concepts
Unit 2	Movement	(2.1) Movement
		(2.2) Velocity
		(2.3) Relation between movement and acceleration
Unit 3	Forces	(3.1) Interactions between bodies
		(3.2) Forces and movement
Unit 4	Rotation and force	(4.1) Particles
		(4.2) Forces and position
Unit 5	Pressure and atmosphere	(5.1) Pressure
		(5.2) Atmosphere
Unit 6	Energy and work	(6.1) Energy
		(6.2) Work
Unit 7	Heat	(7.1) Heat
Unit 8	Movement and undulatory phenomena	(8.1) Types of movement
		(8.2) Undulatory phenomena
Unit 9	Sound	(9.1) Sound
Unit 10	Light and color	(10.1) Light and color
		(10.2) Color

### Procedure

Authorization had previously been requested, at the start of the study, from the educational center and from the Bioethics Committee of the University of Burgos. Subsequently, the parents or legal tutors of the participants were informed, and their informed consent was requested in writing. The instruction was conducted over 28 weeks using the SRL methodology. To do so, the “SRL of physics concepts” of Queiruga et al. (2016) was applied. This methodology consists of presenting questions to the students on physics concepts that were supported by carefully designed images to facilitate conceptual comprehension and SRL; an example by thematic unit can be consulted in the Supplementary Table S1.

The responses of the students were modeled and prompted by the teacher strengthening the correct construction of the concept; an example of the development of SRL learning can be seen in the Supplementary Table S2.

Work proceeded with 10 thematic units, divided into 19 sub-units, the list of which may be consulted in Table 1.

In each thematic sub-unit, an audio file was recorded, amounting to a total of 19 files, which in turn were sub-divided into 509 textual units of observation (conversations between students and the teacher during the curricular instruction). The list of these units of observation by student and by theme can be consulted in Table 2. The units registered by thematic unit were situated within an interval of 8/9. The Van der Stel and Veenman (2014) protocol was used to characterize the responses. Two evaluators assigned the sentences (transcriptions) to their various categories. The size of the units of analysis were decided by looking at the number and the type of ideas expressed by the students. Each unit of analysis had to contain a more or less complete idea.

**TABLE 2:** Number of interventions registered for the dialog by student in each thematic unit.

**Students**	**Interventions**	
	***n***	**Percentage**
1	26	5.1
2	66	12.97
3	71	13.95
4	54	10.7
5	34	6.68
6	56	11
7	46	9.04
8	65	12.77
9	41	8.05
10	50	9.82
Total	509	100

With respect to the categorization process of the metacognitive skills used by the students in the resolution of physics tasks, in the first place, the protocol coded by the first evaluator was taken as the example protocol to illustrate the different categories. Subsequently, the second evaluator marked a second protocol in discussion with the first evaluator. Finally, a third protocol registered the discrepancies in the categorizations. Both the protocols that had been individually completed and the protocol of discrepancies were subsequently jointly analyzed by both evaluators. The Pearson’s contingency coefficient was used to find the reliability index between evaluators for the 19 audio protocols under analysis. The contingency coefficient between the two evaluators was 0.96, so the inter-evaluator reliability may be considered very good, because *C* = 0 indicates independence and *C* = 1 indicates a perfect association between the evaluation criteria of both evaluators.

In addition, the categorization process of the responses given by the students was done using the evaluation structure of the protocols of Veenman et al. (2014) as a reference applied to the area of physics knowledge.

### Data Analysis

The following statistical techniques were used: (a) descriptive statistics (mean, standard deviation, percentages, and frequencies); (b) Crosstab and Pearson’s Chi-Squared test on SPSS (2016 v.24) software; and (c) the Kruskal algorithm was applied for matrices with both a maximum and a minimum value for graph analysis and the Heirholzer algorithm to find the Eulerian Circuit, in both cases using Grafos software v.1.3.5 by Rodríguez-Villalobos (2012).

## Results

### Frequency Analysis of the Type of Responses From Students for the Resolution of Physics Problems Over One Academic Year

With regard to the first objective, the type of response given by the students over one academic year for the resolution of physics problems was studied. In the first place, the responses given by the students were categorized under six headings: Type 1: Does not relate the content that is under study. Type 2: Correct response. Type 3: Lack of vocabulary for a strict definition of the physics concept. Type 4: Arrives at a correct conclusion and relates what is remembered. Type 5: Ambiguous understanding. Type 6: Conceptual error. The categorization was completed by two evaluators and, after applying the alpha coefficient of Hayes and Krippendorff (2007), it yielded an index of 0.896. Scores below 0.70 are considered to tend toward low statistical significance (Krippendorff, 2013). The conclusions should be discounted for variable values of less than 0.67, tentative conclusions may be reached for values between 0.67 and 0.80, and definite conclusions are associated with values above 0.80.

As may be seen from Table 3, the type of response appeared to depend on the concept under consideration. The highest frequency of responses was detected for Type 5 (43/94), which refers to an ambiguous understanding of the physics concepts. This type of response had a higher incidence in Unit 7 (11/43). Subsequently, it was followed by response Type 6, which refers to conceptual errors (20/94) and that had a higher frequency in Unit 4 (13/20). Likewise, Type 3 refers to the difficulties over expressing the physics concept that is considered with an acceptable term, which registered a frequency of 19/94 with a higher index of appearance in Unit 4 (6/19).

**TABLE 3:** Analysis of frequencies and percentages of the type of response given by the students.

**Unit**	**Thematic**	**Type 1**	**Type 2**	**Type 3**	**Type 4**	**Type 5**	**Type 6**	**Total type -unit**	**Percentage**
Unit 1	Scientific methods. Physical magnitudes.	2	3	5	1	5	0	16	17.02
Unit 2	Movement	0	1	0	0	4	0	5	5.32
Unit 3	Forces	3	1	3	0	2	0	9	9.57
Unit 4	Rotation and force	0	0	6	0	3	13	22	23.40
Unit 5	Pressure and atmosphere	0	0	4	0	2	3	9	9.57
Unit 6	Energy and work	0	0	1	0	0	2	3	17.02
Unit 7	Heat	0	0	0	0	11	0	11	11.70
Unit 8	Movement and undulatory phenomena	0	0	0	0	5	0	5	5.32
Unit 9	Sound	0	0	0	0	6	0	6	6.38
Unit 10	Light and color	0	1	0	0	5	2	8	8.51
Total type		5	6	19	1	43	20	94	100
Percentage type error		5.32	6.38	20.21	1.06	45.74	21.28	100.00	

*Type 1, Does not relate the content that is under study; Type 2, Correct response; Type 3, Lack of vocabulary for a strict definition of the physics concept; Type 4, Arrives at a correct conclusion and relates what is remembered; Type 5, Ambiguous understanding; Type 6, Conceptual error.*

In summary, a reduction in the frequency of responses with conceptual errors and uncertain use of vocabulary to express the physics concepts was noted throughout the academic year. Nevertheless, ambiguous understanding of the physics concepts was maintained, and neither was there an increase in correct responses, nor in responses relating to conceptual interrelation.

### Longitudinal Analysis of the Type of Metacognitive Skills in Use and of Their Quality Use^1^

The “Protocol for the analysis of the quality of metacognitive skills” of Van der Stel and Veenman (2014), in this case adapted to the evaluation of the learning of physics concepts, was used to test the second objective of the investigation. As indicated earlier, this instrument is used to analyze quality use of metacognitive skills (Orientation, Planning, Evaluation, and Elaboration) in natural environments. The evaluation criteria ranged from 0 (never uses the strategy) to 4 (always makes the best possible use of the strategy). The reliability indicators of the protocol for this study were high: the general reliability of the instrument was α = 0.84; in relation to the metacognitive skills, its reliability was α = 0.78 for Orientation; α = 0.77 for Planning; α = 0.79 for Evaluation, and α = 0.84 for Elaboration.

In Table 4, the frequency of quality use of metacognitive skills under the criteria (1–4) can be seen. Likewise, in Table 5, the graded levels of significance of each metacognitive skill can be consulted. The highest percentage of use in the Orientation skills and in those of Planning was found at level 3. However, it was at level 1 for the Evaluation and Elaboration skills. No percentages were found at level 0 for any of the metacognitive skills.

**TABLE 4:** Frequency of use: quality use of metacognitive skills for the evaluation criteria.

**Grading of acquisition level (see Table 5)**	**Orientation MS**		**Planning MS**		**Evaluation MS**		**Elaboration MS**	
	**Frequency**	**Percent**	**Frequency**	**Percent**	**Frequency**	**Percent**	**Frequency**	**Percent**
1	73	14.34	109	21.41	307	**60.31**	399	**78.39**
2	140	27.50	138	27.11	102	20.04	72	14.15
3	268	**52.65**	234	**45.97**	93	18.27	38	7.47
4	28	5.50	28	5.50	7	1.38	0	0
Total	509	100	509	100	509	100	509	100

*MS, metacognitive skills. The graded level of significance can be consulted in Table 5. The highest percentages are shown in bold type.*

**TABLE 5:** Grading of acquisition level.

**Skill type**	**Acquisition level**				
	**0**	**1**	**2**	**3**	**4**
Orientation Metacognitive Skills	Never uses the strategy	Centers on the task (objective)	Asks superficial questions with respect to the objective of the task	Applies the strategy both to the object of the task and to the previous knowledge needed to resolve it	Uses the strategy in a reflexive way in relation to the resolution of the task
Planning Metacognitive Skills	Never uses the strategy	No plan of action is observed for the resolution of the task	Uses test-error strategies	Systematic steps are not followed in resolving the task	Follows a carefully designed plan
Evaluation Metacognitive Skill	Never uses the strategy	No comprehension of the task is observed	Self-corrects errors	Performs monitoring of the evaluation through questions during problem-solving	Completes a final evaluation of the resolution process
Elaboration Metacognitive Skills	Never uses the strategy	Occasionally reflects at times on the objective of the task and the responses needed for its resolution	Conducts systematic reflection both on the objective of the task and on the responses for its solution	Conducts systematic reflection both on the objective of the task and on the responses for its resolution and identification of the most significant conclusions	Reflects on how learning has taken place and on generalization strategies for other tasks
					

### Longitudinal Analysis of the Quality Use of the Metacognitive Skills of Each Student

The quality use of metacognitive skills (in keeping with the definition of Veenman and Spaans, 2005; Van der Stel and Veenman, 2014) was analyzed. In the first place, data analysis was performed, applying radial graphs by thematic unit over one academic year (see Supplementary Figure S1). Different patterns were found for the quality use of metacognitive skills in the different thematic units. Likewise, differences in the patterns of use of the metacognitive skills were observed, even in a single student in the same thematic unit. As may be observed from Supplementary Figure S1, the type and quality use of each metacognitive skill varied among the students, even over the same thematic unit. Subsequently, two Crosstab analyses were completed, with a view to testing the relation between quality use of metacognitive skills and the variables. In the first analysis, the variable students and the variable quality use of metacognitive skill were both cross tabbed. As may be seen in Table 6, the level of quality with the greatest frequency of use among the metacognitive skills was level 3 (52.65%), which implies a relation between the end-purpose of each task and previous knowledge of the subject. With regard to the metacognitive skill of Planning, level 3 showed a higher frequency of use (45.97%), which implies the development of a non-systematic problem-solving plan. With regard to the metacognitive skill of Evaluation, level 1 was used with the highest frequency (60.31%), which implies no use of systematic actions of evaluation. With regard to the metacognitive skill of Elaboration, the level of quality with the greatest frequency of use was level 1 (78.39%), which indicates that the students occasionally related the end-purpose of the task with the responses that they gave for its solution. Likewise, significant differences were found for quality use of all the metacognitive skills (Orientation χ^2^ = 227.63 *p* = 0.000; Planning χ^2^ = 135.03, *p* = 0.000; Evaluation χ^2^ = 161.29, *p* = 0.000; Elaboration χ^2^ = 59.49 *p* = 0.000) among the students. It implies that there was no homogeneity in the quality of all the metacognitive skills among the students and that different learning patterns existed in the same group.

**TABLE 6:** Crosstab between the variable students and Grading of acquisition level in metacognitive skills.

**MS**	**Grading of acquisition level**	**Students**										**Total**	**%**	**χ^2^**
		**1**	**2**	**3**	**4**	**5**	**6**	**7**	**8**	**9**	**10**			
Orientation	1	12	0	8	3	12	9	13	5	4	7	73	14.34	227.63^∗^
	2	0	15	4	58	2	4	2	18	20	17	140	27.50	
	3	1	47	9	78	4	7	1	42	35	44	268	**52.65**	
	4	2	7	0	7	1	1	0	5	3	2	28	5.50	
Planning	1	12	7	8	17	12	10	13	6	8	16	109	21.41	135.03^∗^
	2	0	18	4	54	2	4	2	22	18	14	138	27.11	
	3	1	37	9	68	4	6	1	37	33	38	234	**45.97**	
	4	2	7	0	7	1	1	0	5	3	2	28	5.50	
Evaluation	1	13	39	16	85	14	15	15	35	32	43	307	**60.31**	61.29^∗^
	2	2	19	4	31	0	4	1	24	14	3	102	20.04	
	3	0	10	1	27	4	1	0	11	15	24	93	18.27	
	4	0	1	0	3	1	1	0	0	1	0	7	1.38	
Elaboration	1	14	59	19	113	14	19	16	55	44	46	399	**78.39**	59.49^∗^
	2	1	10	1	23	2	2	0	13	14	6	72	14.15	
	3	0	0	1	10	3	0	0	2	4	18	38	7.47	

*^∗^*p* < 0.005. MS, metacognitive skills. 1, 2, 3, and 4, see Table 5 for the interpretation of Grading of acquisition level. The highest percentages are shown in bold type.*

In summary, quality use of metacognitive skills among the students followed a very similar path for the metacognitive skills of both Orientation and Planning and the level of acquisition was at level 3. Likewise, a common path was observed for the use of the metacognitive skills of Evaluation and Elaboration, where acquisition was at level 1.

With regard to the quality use of metacognitive skills for the different thematic units, the same percentages were found as seen earlier [in the Orientation metacognitive skills the level of acquisition was 3 (52.65%)], the same occurred for the Planning metacognitive skills (level 3 = 45.97%). Nevertheless, the use of the Evaluation and Elaboration metacognitive skills were situated at level 1 (60.31 and 78.39%, respectively), see Table 7.

**TABLE 7:** Crosstab between the variable students and Grading of acquisition level in metacognitive skills.

**MS**	**Grading of acquisition level**	**Unit**										**Total**	**%**	**χ^2^**
		**1**	**2**	**3**	**4**	**5**	**6**	**7**	**8**	**9**	**10**			
Orientation	1	6	14	6	15	4	11	4	3	3	7	73	14.34	210.72^∗^
	2	9	23	39	14	19	4	2	19	0	11	140	27.50	
	3	1	26	23	25	6	41	40	36	38	32	268	**52.65**	
	4	10	3	3	0	5	0	0	7	0	0	28	5.50	
Planning	1	7	34	19	16	4	12	4	3	3	7	109	21.41	228.38^∗^
	2	9	18	27	13	19	18	2	19	1	12	138	27.11	
	3	0	11	22	25	6	26	40	36	37	31	234	**45.97**	
	4	10	3	3	0	5	0	0	7	0	0	28	5.50	
Evaluation	1	25	66	71	54	34	13	6	18	3	17	307	**60.31**	558.19^∗^
	2	1	0	0	0	0	42	29	15	1	14	102	20.04	
	3	0	0	0	0	0	1	11	25	37	19	93	18.27	
	4	0	0	0	0	0	0	0	7	0	0	7	1.38	
Elaboration	1	26	66	71	54	34	50	35	32	4	27	399	**78.39**	603.79^∗^
	2	0	0	0	0	0	5	11	33	0	23	72	14.15	
	3	0	0	0	0	0	1	0	0	37	0	38	7.47	

*^∗^*p* < 0.005. MS, Metacognitive Skills. 1, 2, 3, and 4, see Table 5 for the interpretation of Grading of acquisition level. The highest percentages are shown in bold type.*

### Analysis of the Pattern of Use of the Metacognitive Skills of Each Student

A personalized analysis was also conducted on the patterns of use of metacognitive skills for each student, for which purpose the graph analysis technique was applied. In the first place, the matrices were found with both the maximum and the minimum frequencies registered for each student in each of the thematic units (see Tables 8, 9). As can be seen, the frequencies of use have a broad spectrum of variability between the students. With regard to the frequency matrix of minimum values, the students for whom higher frequencies were found were (4, 10, 9, 8, and 2) and the interval of frequencies fluctuated between 76 and 192. With regard to the frequency matrix of maximum values, the students for whom very high values were found were 4, 10, 2, 8, and 9. In this case, the interval of frequencies fluctuated between 94 and 339. It is important to point out that different patterns of behavior were registered with regard to the use of the metacognitive skills among the students. Students 4 and 10 presented higher frequencies for the use of the metacognitive skills, both for the minimum and for the maximum values, for which reason it could be said that they have a constant pattern of learning. However, other students, such as student 2, who started with low values in the use of metacognitive skills continued to increase and to improve their use throughout the thematic units, finally reflecting a profile of progressive improvements in the quality of their metacognitive skills. There is also another profile of students (for example, 1 and 7) who always registered minimum or null values in the quality of their metacognitive skills. This type of academically challenged student could be at risk of academic failure and/or leaving the course.

**TABLE 8:** Frequency matrix of minimal use of the metacognitive skills of each student from greater to lesser frequency.

**Students**	**Thematic units**										
	**1**	**2**	**3**	**4**	**5**	**6**	**7**	**8**	**9**	**10**	**Total**
**4**	0	15	26	16	12	11	12	49	30	17	192
**10**	0	16	8	9	5	2	9	1	54	7	121
**9**	0	9	8	6	5	2	9	17	12	14	91
**8**	0	1	2	8	2	25	14	8	3	9	80
**2**	7	2	14	5	4	9	8	10	1	14	76
5	0	2	1	2	1	2	1	4	9	3	30
6	0	2	6	2	1	4	1	4	1	2	29
3	0	0	4	2	2	4	1	3	3	2	24
7	0	3	1	2	1	2	1	1	1	2	21
1	2	2	1	2	1	2	1	1	1	3	17

*The highest percentages are shown in bold type.*

**TABLE 9:** Frequency matrix of maximum use of metacognitive skills of each student from greater to lesser frequency.

**Students**	**Thematic units**										
	**1**	**2**	**3**	**4**	**5**	**6**	**7**	**8**	**9**	**10**	**Total**
**4**	0	37	61	41	1	26	12	94	30	33	339
**10**	0	39	18	21	13	2	18	1	54	11	187
**2**	21	2	38	15	11	27	24	19	1	22	182
**8**	0	29	4	17	5	57	24	13	3	19	179
**9**	0	20	18	12	13	4	27	29	12	21	165
3	0	0	9	4	29	10	29	5	3	2	94
6	0	2	14	2	1	10	1	7	1	4	48
5	0	2	1	2	1	2	1	7	9	5	35
7	0	4	1	2	5	2	1	1	1	4	28
1	8	2	1	2	1	2	1	1	1	4	24

*The highest percentages are shown in bold type.*

**FIGURE 1:**
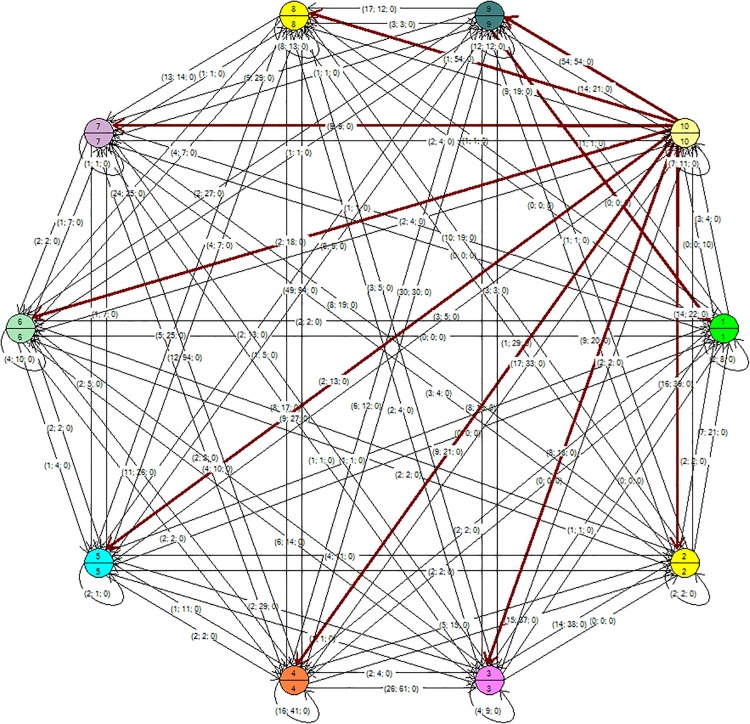
Minimum value tree generated with the Kruskal algorithm. Note: the node has two dimensions: the variable student and the variable thematic unit. The connecting arrows underlined in red indicate the relations of the student in the thematic unit.

**FIGURE 2:**
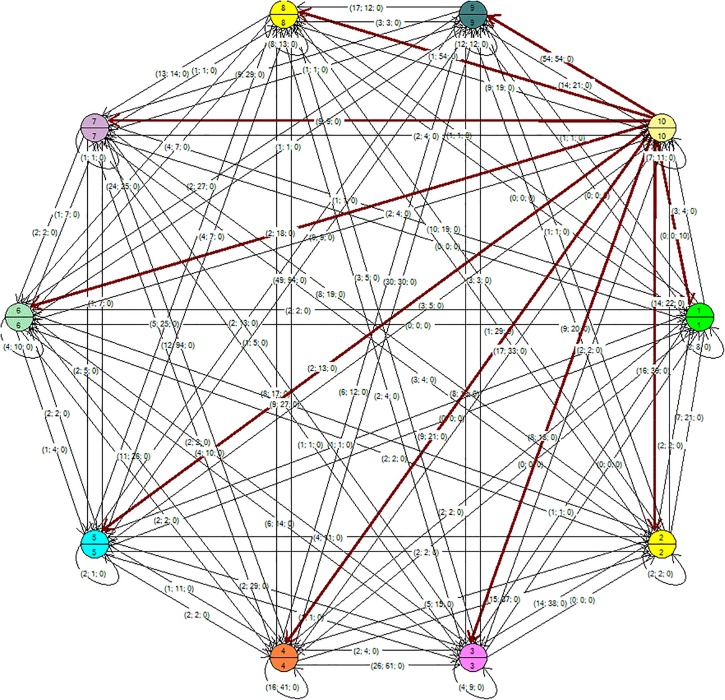
Maximum value tree generated with the Kruskal algorithm. Note: the node has two dimensions: the variable student and the variable thematic unit. The connecting arrows underlined in red indicate the relations of the student in the thematic unit.

**FIGURE 3:**
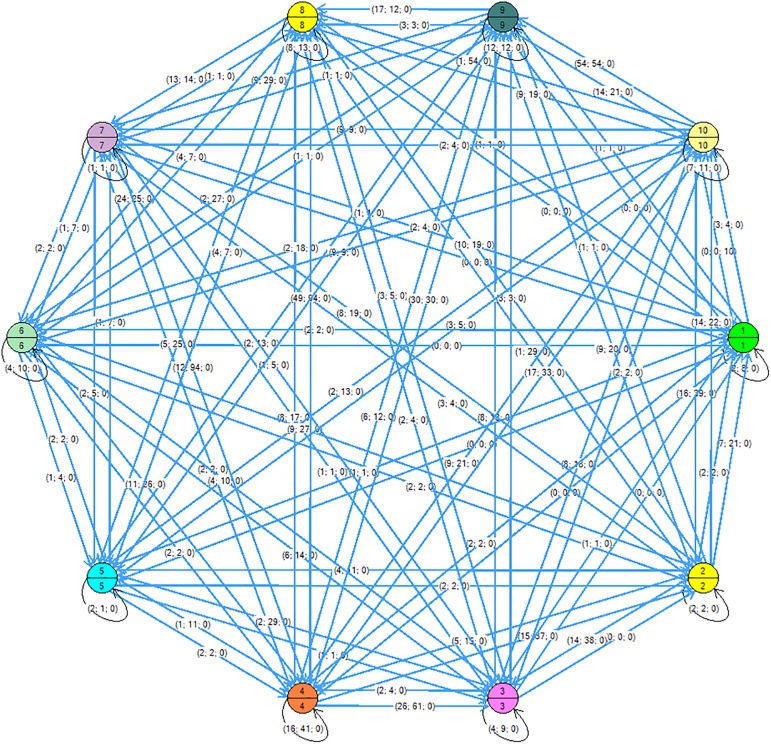
Eulerian circuit generated with the Heirholzer algorithm.

In Figures 1, 2, the tree may be seen with the maximum and the minimum values of a single student for each of the thematic units (represented in the node). The tree was generated with the Kruskal algorithm at minimum values (a minimum expansion tree is a tree composed of all the vertices and the sum of its edges has the lowest weight) and at maximum values (the sum of the edges has the highest weight). The general graph on the Eulerian plane was also found, for which the Heirholzer algorithm was used (the Eulerian circuit is a closed path that passes along each edge only once; a graph has a Eulerian cycle if it is connected and each vertex is of even degree), see Figure 3. Both algorithms were found with the Grafos software tool (Rodríguez-Villalobos, 2012).

## Discussion and Conclusion

The conceptual understanding of physics concepts and its correct application to the resolution of physics problems appears to show no uniform behavior. Instead, correct understanding appears to depend on two factors: on the one hand, the type of physics concept (Pozo, 1994; Queiruga et al., 2016) and, on the other hand, the type of learning patterns of each student (Sáiz and Queiruga, 2018). The difficulties found with this study are defined by ambiguous responses, conceptual errors, and uncertain vocabulary for a rigorous expression of each physics concept. However, a reduction in conceptual errors and less uncertainty over the correct vocabulary was noted over the course of the academic year, despite the persistence of ambiguous conceptual understanding. One possible explanation is that learning physics implies the construction of concepts that have a high component of abstraction, which means that their understanding is complex for the student (Pozo, 1994). Likewise, the hypothesis that comprehension is conditional upon previous knowledge and the learning style of the student (Taub and Azevedo, 2019) will be tested in future investigations.

In contrast, use of the different metacognitive skills (Orientation, Planning, Evaluation, and Elaboration) in a homogeneous manner was not observed. A greater use of the metacognitive skills of Orientation and Planning, as against Evaluation and Elaboration, was found. Likewise, quality use or the degree of achievement of the first two metacognitive skills was greater than for the last two. Those results are related with the activities that imply the use of certain sorts of metacognitive skills. The first refer to orientation activities for task resolution and planning of problem-solving strategies, and the second imply more complex cognitive and metacognitive processes such as supervision and evaluation of task completion (Veenman and Beishuizen, 2004; Azevedo et al., 2011; Schellings et al., 2013; Van der Stel and Veenman, 2014; Sáiz and Montero, 2015; Sáiz and Marticorena, 2016; Veenman and Van Cleef, 2019). These conclusions are relevant for the preparation of programs of intervention in the field of science teaching and STEM (Science, Technology, Engineering, and Mathematics) materials. Programs based on SRL techniques (Núñez et al., 2011; Taub and Azevedo, 2019) will especially be analyzed in future studies, to find out whether this type of intervention increases the use of the metacognitive skills of Evaluation and Elaboration in the learning of STEM materials.

Likewise, different patterns of student learning behaviors were found. It can therefore be concluded that the use of on-line methods and techniques of analysis of learning patterns such as graph analysis will give the teacher personalized information on the development of each student (Sáiz et al., 2019). This is a key aspect for the design of personalized learning programs and for the identification of academically challenged students at risk of dropping out (Reoyo et al., 2017). Future research will center on studying the characteristics of effective versus ineffective learning patterns for the learning of STEM subjects.

In summary, it is relevant to note that the use of an observational methodology has proven its effectiveness as a tool for individualized follow up of the learning process among students (Johnson et al., 2007; Anguera et al., 2018a) specifically in those that use on-line registers (Veenman et al., 2014; Veenman and Van Cleef, 2019). The use of this type of technique permits longitudinal and personalized follow up. The information facilitated error analysis that opens the door to personalized follow-up of each student. Likewise, the application of the liquefying technique to the registers of the sequence of events facilitated systematic analysis of the behaviors and the application of quantitative methods in the analysis of the results of the observation (Anguera and Hernández-Mendo, 2016; Anguera et al., 2017a; Rodríguez-Medina et al., 2018). As a result, it may be concluded that this methodology has facilitated an exhaustive and personalized analysis of learning patterns. However, its use was limited to small samples and involved the application of registry techniques and transformational analysis of data that, despite their improvement with the release of new software, continue to have significative costs in terms of registry time, transformation, and processing of the data for the investigator (Anguera et al., 2017b). However, the generalization of the results of this study must therefore be done with prudence, due to the characteristics of the sample (size and origin). Nevertheless, the use of on-line evaluation techniques of metacognitive skills yields a personalized study of the quality use of those skills, a procedure that would be difficult to approach with very large samples. In addition, this type of methodology increased the ecological validity of the results (Anguera et al., 2018a). However, as has been pointed out, they have a high cost in terms of time and personal resources, because they have to involve at least two teachers in the process of mixed observation, in order to be able to establish the indicators of reliability for the classification of the registers (Veenman, 2011; Schellings et al., 2013; López-Roldán and Fachelli, 2015).

## Data Availability Statement

The datasets generated for this study are available on request to the corresponding author.

## Ethics Statement

The Ethics Committee of the University of Burgos approved this study. Written informed consent was in each case requested from the parents and, where applicable, the legal guardians of the participating students. They all gave their written informed consent in accordance with the Declaration of Helsinki.

## Author Contributions

MS performed the statistical and data mining analyses and data interpretation and prepared the manuscript. MQ prepared and set in motion the SRL program for the learning of physics. CG**-**O supervised the application of data-mining techniques in the observational matrix. EM supervised the physics contents of the SRL program. JR**-**M supervised the observational design and the graph analyses.

## Conflict of Interest

The authors declare that the research was conducted in the absence of any commercial or financial relationships that could be construed as a potential conflict of interest.

## Footnotes

1 According to the definition of Veenman and Spaans (2005) and Van der Stel and Veenman (2014).
