# Supplementary material for: Observation of Metacognitive Skills in Natural Environments: A Longitudinal Study With Mixed Methods
Source: Front Psychol. 2019 Nov 1;10:2398. doi: 10.3389/fpsyg.2019.02398 (PMC6838136; doi:10.3389/fpsyg.2019.02398)
Supplement: Supplementary file 1 [file Data_Sheet_1.docx]

Supplementary Material

**Supplementary Table S1.** Examples of the use of images and questions for prompting reflection to facilitate SRL and comprehension of the concepts of the “Program of Self-regulated learning of physics concepts” for the 4th year of Secondary Education (Queiruga and Sáiz, 2016).

| Unit 1. Scientific method. Physical magnitudes. | Unit 6. Energy and work |
| --- | --- |
| 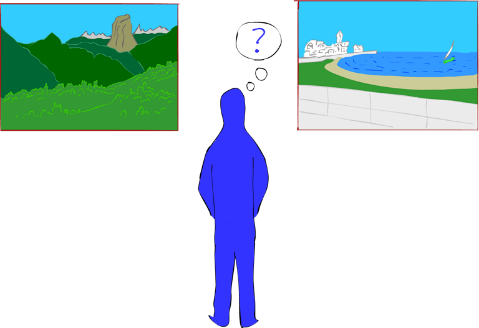 | 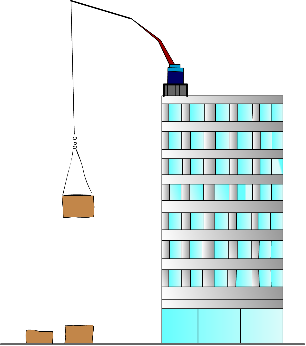 |
| Which of the images is the most beautiful? Try to give a scientific answer! | The bricks have a different potential energy when they are on the floor and when they are on flat roof of the building. What has caused this increase in potential energy? |
| Unit 2. Movement | Unit 7. Heat |
| 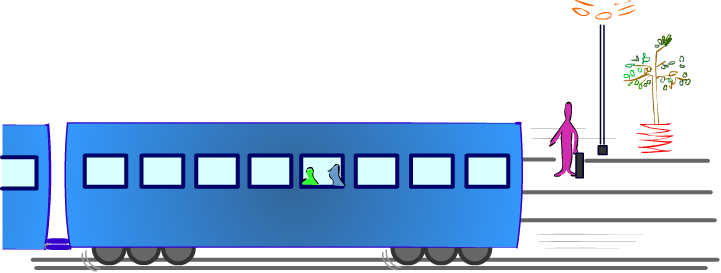 | 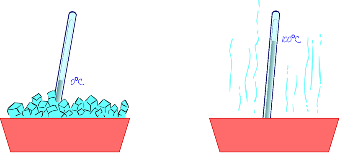 |
| Is it the train or the platform that is moving? | Would you be able to define your own temperature scale? |
| Unit 3. Forces | Unit 8. Movement and undulatory phenomena |
| 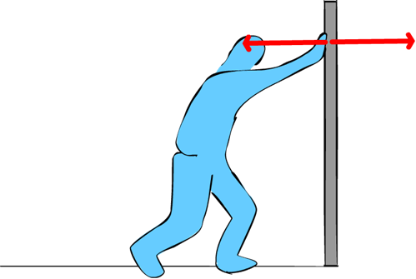 | 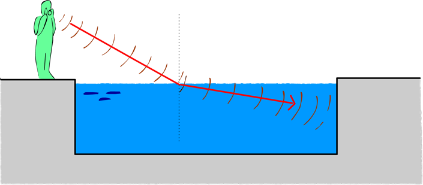 |
| Is the force exercised against the wall equal to the force that the wall exercises on me? | Part of the wave travels through the surface. Does it stay in the same medium? |
| Unit 4. Rotation and force | Unit 9. Sound |
| 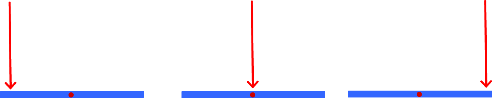 | 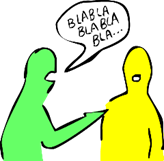 |
| Do the following experiment: take a pencil, hold it in the middle, and apply actions like those in above figure. In what direction will it turn? | A conversation in a normal tone of voice. How many dB will it have? |
| Unit 5. Pressure and atmosphere | Unit 10. Light and Color |
| 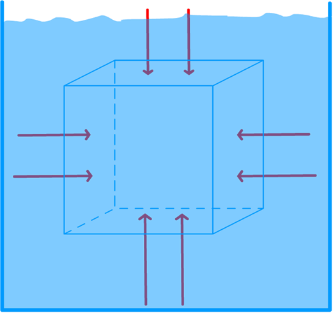 | 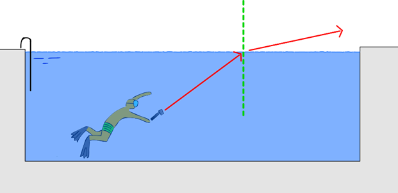 |
| If we place a box inside a liquid, is the force due to the pressure perpendicular to the surface of each side? | Suppose that we are diving in the swimming pool with a torch and we direct the torchlight towards the surface of the water. What might happen to the light? |

**Supplementary Table S2.** Example of a transcription of dialogues and the categorization of the answers of the students using as a reference the protocol for the analysis of the quality of Metacognitive Skills by Van der Stel and Veenman (2014).

| **TASK** | **STUDENTS QUESTIONS** | **TYPE OF ERROR** | **RESPONSES FROM THE TEACHR** | **CATEGORIZATION OF THE RESPONSES FROM THE STUDENTS** | | | |
| --- | --- | --- | --- | --- | --- | --- | --- |
|  |  |  |  | **MO** | **MP** | **MEV** | **MEL** |
| **Definition of the elements**  Beam. Incident beam. Reflected beam. Screen. Image. | Student 8: Why don’t you see the laser? | Unrelated to the content under study. | As the height of the focus varies, the height of the image varies.  What do we see through the lens? The light that is reflected. There have to be particles that reflect the light. | 1 | 1 | 0 | 0 |
|  | Student 2: Why can you see the laser beam in the dark? | Unrelated to the content under study. | The small particles of dust suspended in the air reflect the light of the laser. | 1 | 1 | 0 | 0 |
| 1. Observation of the event on repeated occasions. | Student 1: The angle of impact and reflection are the same. | Correct. The student recalled the lesson and answered correctly. | The majority have not seen this conclusion. The topic is guided back to the reasoning of the heights. | 3 | 3 | 0 | 0 |
| 2. Completion of measurements and data collection. | Student 10: I think that it depends on the angle at which the light impacts.  Student 10: It is always equal on the other side. |  | Can someone draw it? When I shine the beam towards the mirror ¿this…?  Equal? What are you referring to? | 3 | 3 | 0 | 0 |
| 3. Preparation of simplified models or forms that help their comprehension. | Student 2: The same height. | Right. |  | 3 | 3 | 0 | 0 |
|  |  |  | We now have a physics law, don’t we? Is this always going to happen? |  |  |  |  |
|  | Everyone: Yes, it’ll be done.  Student 8: And if you move the laser pointer further away? |  | We can see that if I move the pointer away... this “law of heights” no longer applies …  If we want to make a more general law that doesn’t depend on distances …  We follow the “normal” line”.  What another is law fulfilled? | 3 | 3 | 2 | 1 |
|  | Student 1: The incident angle is equal to the angle of reflection. |  | Now we have another more general law. | 3 | 3 | 2 | 0 |
| 4. Formulation of laws capable of explaining similar problems to the last one. | Student 2: What is that law called? |  | The law of reflection. | 3 | 3 | 2 | 0 |

Note. O = Metacognitive Skills of Orientation; P = Metacognitive Skill of Planning; MEV = Metacognitive Skills of Evaluation; MEL = Metacognitive Skills of Elaboration.

MO: 0 = Never uses the skill; 1 = Centers on the task (objective); 2 = Asks superficial questions with respect to the objective of the task; 3 = Applies the skill to the relation between the object of the task and the previous knowledge needed to resolve it; 4 = Uses the skill in a reflexive way in relation to the resolution of the task; MP: 0 = Never uses the skill; 1 = No plan of action is observed in the resolution of the task; 2 = Uses test-error strategies; 3 = Systematic steps are not followed in resolving the task. 4 = Follows a carefully designed plan;

MEV: 0 = Never uses the skill; 1 = No comprehension of the task is observed; 2 = Self-corrects errors; 3 = Performs monitoring of the evaluation through questions during problem-solving; 4 = Completes a final evaluation of the resolution process.

MEL: 0 = Never uses the skill; 1 = Conducts occasional reflection between the objective of the task and responses for solving the problem; 3 = Conducts systematic reflection on both the objective of the task and the problem-solving responses and determines the most significative conclusions. 4 = Reflects on how learning has taken place and on generalization skill for other tasks.

**Figure S1.** The patterns of use of the metacognitive skills in each thematic unit, applying radial graphs.

| 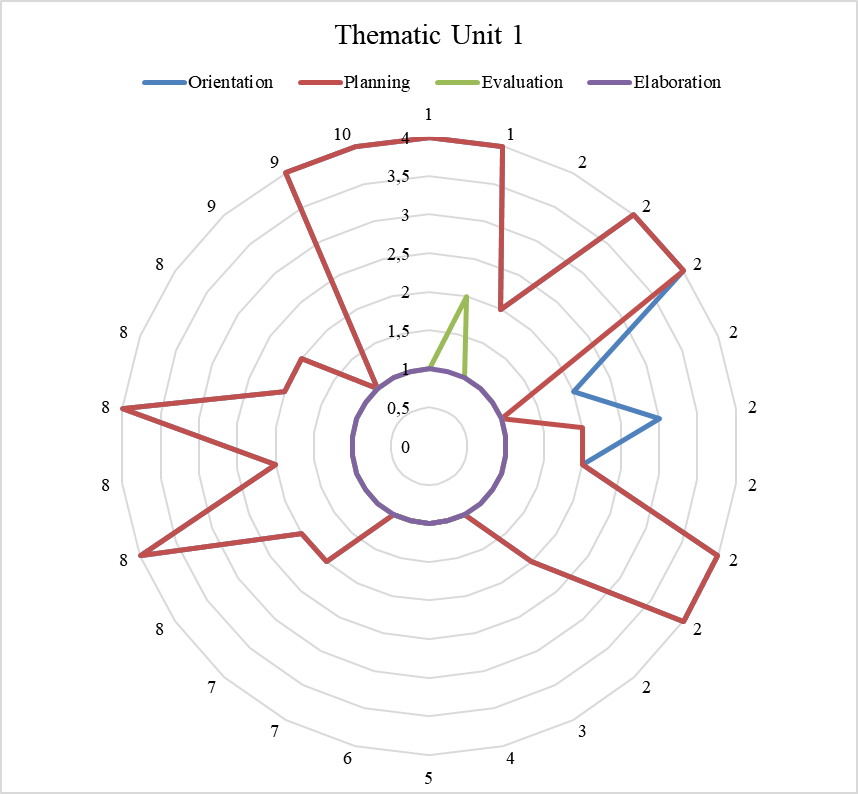 | 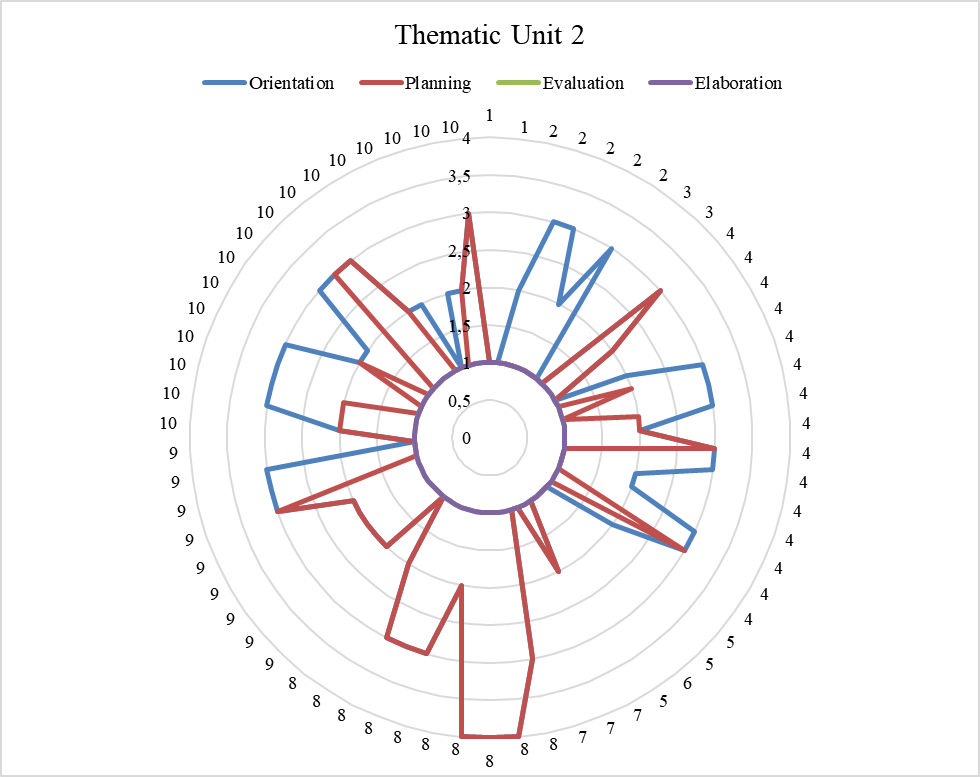 |
| --- | --- |
| 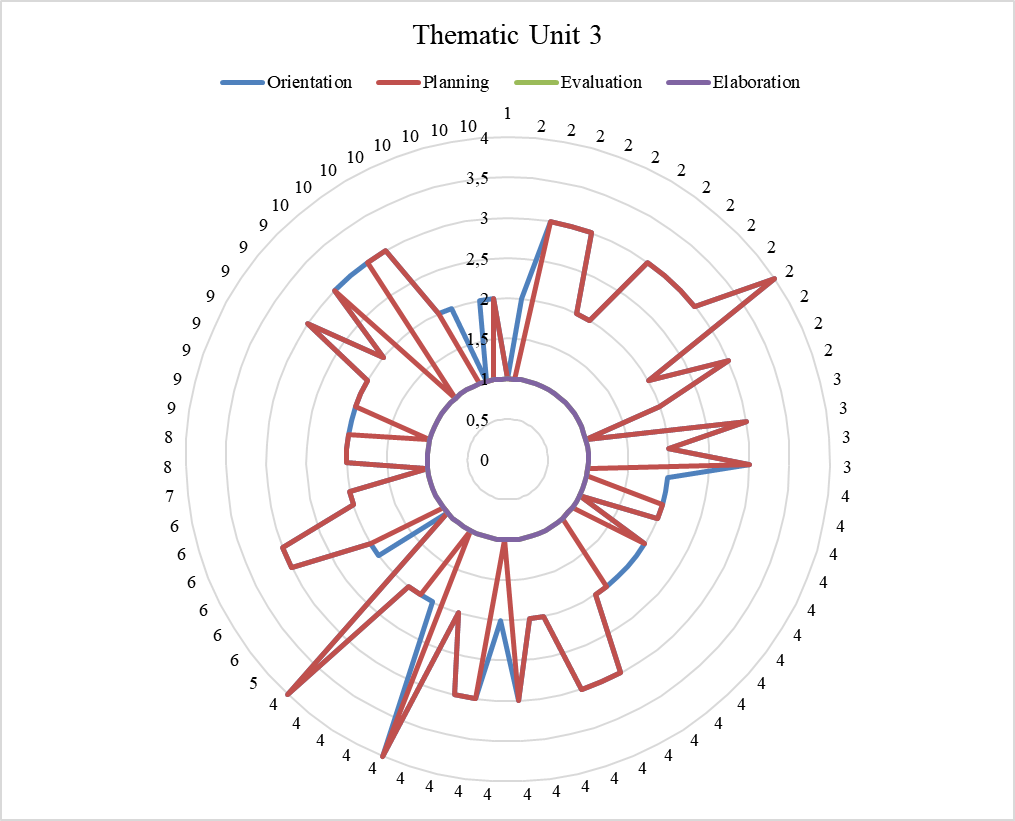 | 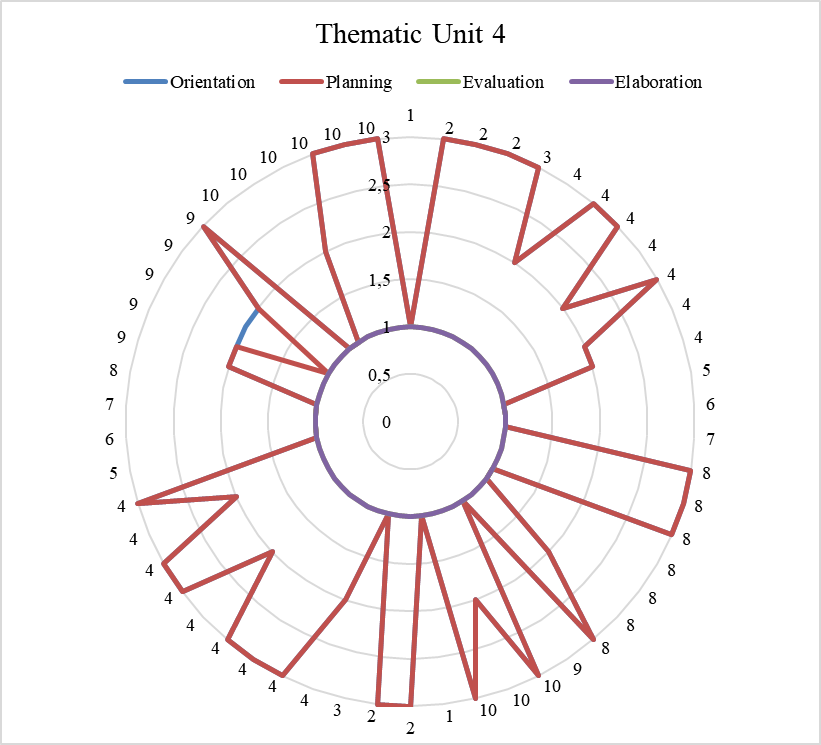 |
| 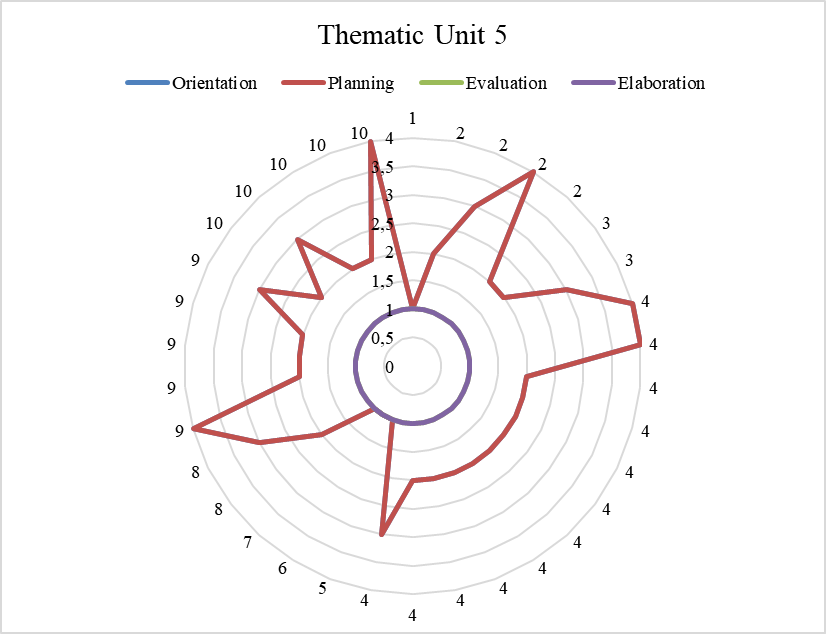 | 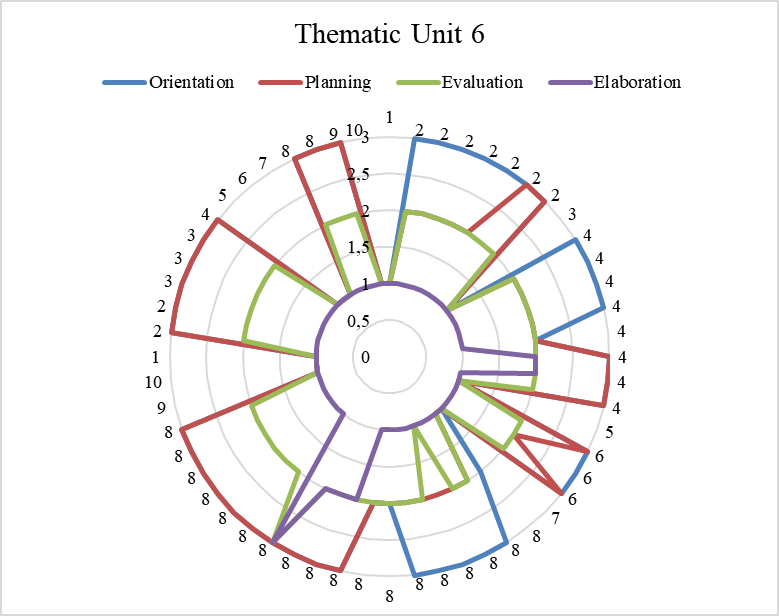 |
| 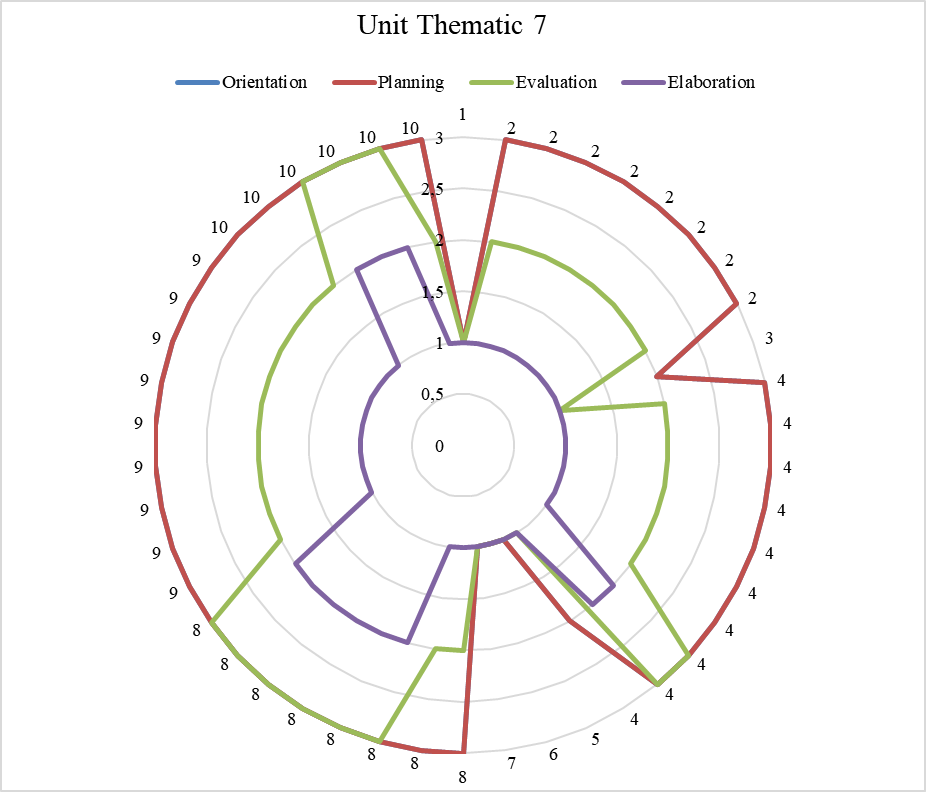 | 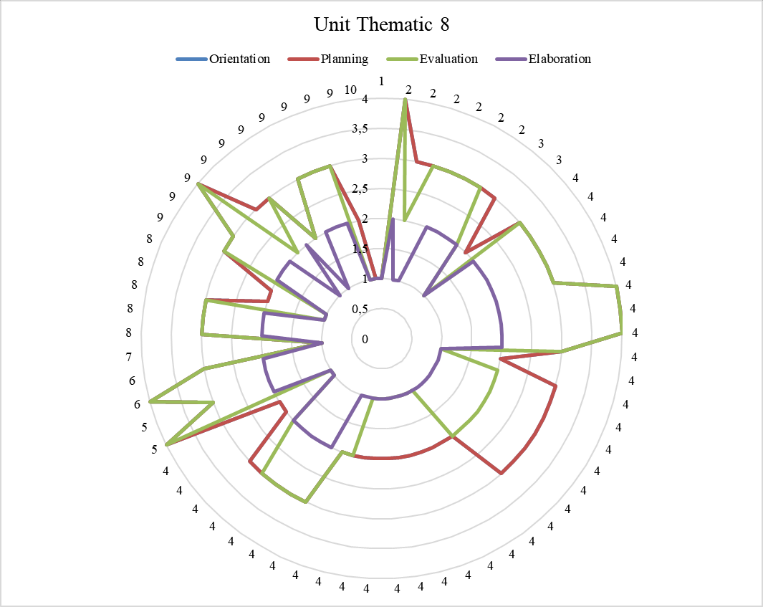 |
| 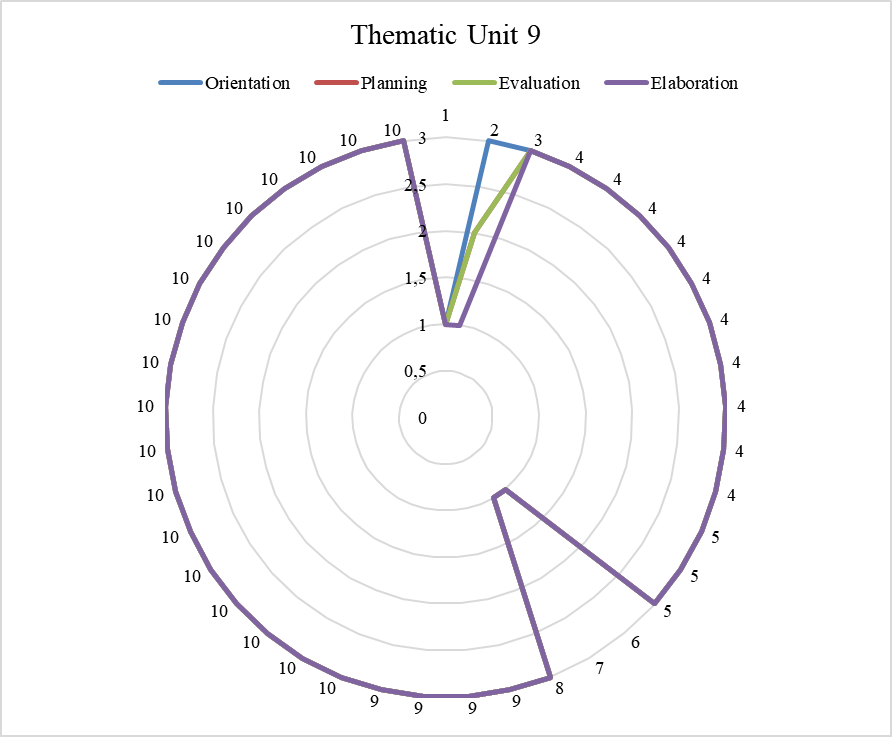 | 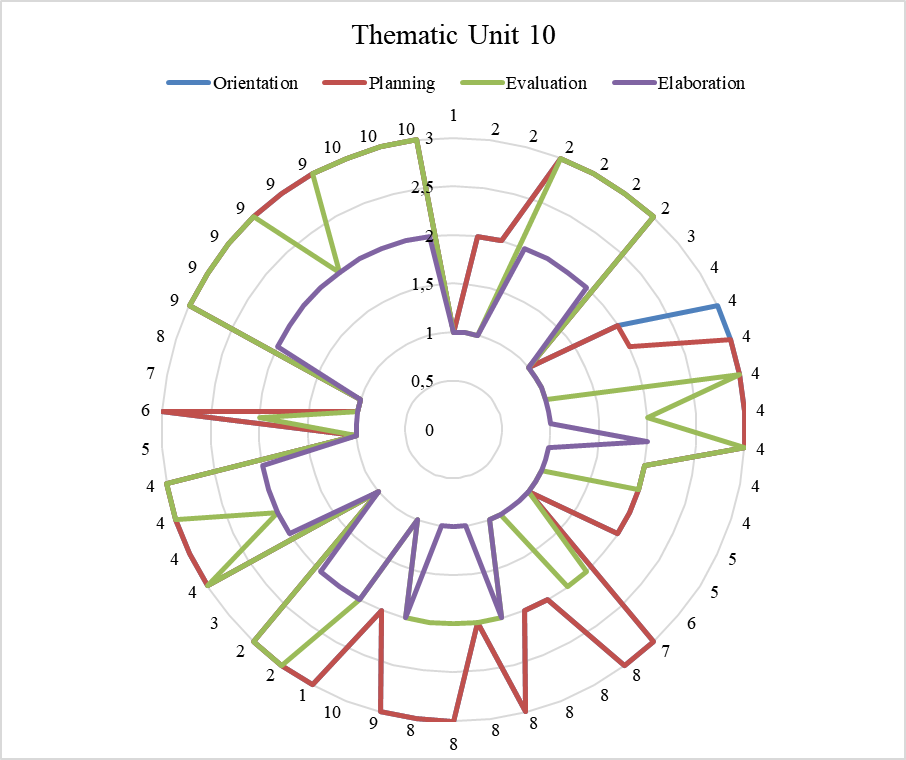 |

Note. 1 = student 1, 2 = student 2, 3 = student 3,4 = student 4, 5 = student 5, 6 = student 6, 7 = student 7, 8 = student 8, 9 = student 9, 10 = student 10.
